# Psychometric properties of the Norwegian version of the Kidscreen-27 questionnaire

**DOI:** 10.1186/s12955-016-0460-4

**Published:** 2016-04-09

**Authors:** John Roger Andersen, Gerd Karin Natvig, Kristin Haraldstad, Turid Skrede, Eivind Aadland, Geir Kåre Resaland

**Affiliations:** Department of Health Studies, Sogn og Fjordane University College, Svanehaugvegen 1, 6812 Førde, Norway; Centre of Health Research, Førde Hospital Trust, Førde, Norway; Department of Global Public Health and Primary Care, University of Bergen, Bergen, Norway; Department of Health and Nursing Sciences, University of Agder, Kristiansand, Norway; Faculty of Teacher Education and Sport, Sogn og Fjordane University College, Sogndal, Norway

**Keywords:** Children, Health-related quality of life, Quality of life, Kidscreen-27, Norway, Reliability, Validity

## Abstract

**Background:**

The Norwegian version of the Kidscreen-27, a measure of generic health-related quality of life, has not yet been validated. Thus, the aim of this study was to investigate the reliability and validity of the Norwegian Kidscreen-27, in 10 year-old children.

**Methods:**

The Kidscreen-27 consists of five domains and was validated in a cross-sectional study of 1085 school children (52.5 % boys). In addition a subsample of 56 children also had repeated measures in order to study test-retest reliability.

**Results:**

Cronbach's alpha values ranged from 0.73 to 0.83, while intraclass correlation values over time ranged from 0.71 to 0.81. The domains of physical well-being, psychological well-being and autonomy & parents improved over time (*Ps* < 0.05), while social support and school environment domains did not. Confirmatory factor analysis showed an acceptable overall model fit: *X*^2^ = 707; *df* = 310; *P* <0.001, root mean squared error of approximation = 0.037, the comparative fit index = 0.96 and the Tucker-Lewis index = 0.95. All factor loading were > 0.40. The Kidscreen-27 domains were significantly associated with general life satisfaction as measured with the Cantrils Ladder (Spearman rank correlations ranged from 0.29 to 0.59, *Ps* < 0.05).

**Conclusion:**

The Norwegian version of Kidscreen-27 has good reliability and validity.

**Electronic supplementary material:**

The online version of this article (doi:10.1186/s12955-016-0460-4) contains supplementary material, which is available to authorized users.

## Background

The Kidscreen-27 is a well-validated, short, multidimensional measure of generic health-related quality of life (HRQoL) in children and adolescents which is available in many languages [1, 2]. However, unlike the longer Kidscreen-52, the Norwegian version of the Kidscreen-27 has not yet been validated [3]. The Kidscreen-27 is a particularly useful instrument for assessing HRQoL in younger children as a consequence of its content, ease of completion and the volume of available data with which international comparisons can be made. As our research group has planned several studies on HRQoL in younger children, the aim of this study was to investigate the reliability and validity of the Norwegian Kidscreen-27 questionnaire in 10-year-old children.

## Methods

We applied both a cross-sectional and a prospective design in this study, which was approved by the regional ethical research committee (2013/1893, 2012/1089). We recruited children aged 10 years from 52 schools in Western Norway for the cross-sectional study, whereas children from one school were recruited for the prospective study. Written informed consent was obtained from the parents or guardians of the participants. The children were given brief oral information about the questionnaire by their teacher (the information was identical with the standardised information on the first pages of the Kidscreen-27 questionnaire), and then completed it while they sat at their desks in the classroom. After doing so, they were told to carry on with their school work in order to minimize any noise until all the other children were finished. They were allowed to ask the teacher for help if they did not understand any of the questions. Children with reading difficulties were helped by a teaching assistant, as would be the case normally. For a subsample of the children the Kidscreen-27 was administered at three time points: at the beginning of the school day (Test 1); at the end of the same school day (Test 2); and during the middle of the following school day (Test 3).

### Kidscreen-27

Items of the Kidscreen-27 are derived from the Kidscreen-52 questionnaire [2]. It has five domains: physical well-being (5 items); psychological well-being (7 items); autonomy & parents (7 items); social support & peers (4 items); and school environment (4 items). We used the methodology given in the developers manual to obtain the T-scores; mean (±SD) scores of 50 ± 10 define normality for children and adolescents aged 8-18 years across Europe [2]. Higher scores indicate a better HRQoL. The Kidscreen-27 is standardized so that a difference of <0.2 points is considered trivial, 2.0 – 4.9 as small, 5.0–7.9 as moderate and ≥8 as large effects [2, 4]. There are two official forms of written Norwegian, Bokmål and Nynorsk. They are very similar, and it is mandatory that they are both taught in Norwegian schools. The Kidscreen-27 items are available in Bokmål [3], and has been translated from English into Norwegian in accordance with respected guidelines [5]. We decided to use Nynorsk in this study. The linguistic skills required to produce a Nynorsk version of Kidscreen-27 from the Kidscreen-52 are minimal. The translation was undertaken by the first three authors and modified after discussion with a professor of Norwegian languages (see acknowledgements). The final version was based on consensus and no problematic or difficult issues were noted. This process was approved by the European Kidscreen Group.

### Cantrils life satisfaction ladder

In order to study convergent validity we assessed general life satisfaction using the adapted version of the Cantrils Life Satisfaction Ladder. This measure has been used in World Health Organisation surveys of children and adolescents, including in Norway [6]. The child is presented with a picture of a ladder with steps ranging from 0 to 10. They are told that the top step (10) represents the best possible quality of life, while the bottom (0) represents the worst. They are asked to indicate where on the ladder they currently consider themselves to be. This question was assessed together with the Kidscreen-27 in a subsample of the children.

### Statistical analysis

Internal consistency was assessed by calculating Cronbach’s alpha values; values ≥0.7 were considered satisfactory [1, 2]. Floor and ceiling effects were demonstrated by the percentages of children with the lowest and highest possible scores. Test-retest reliability (Tests 1–3) was assessed by calculating single measures intraclass correlation coefficients (ICC), using a two-way mixed model with an absolute agreement definition; ICC values ≥0.7 were considered satisfactory [1, 2]. A linear mixed model based on restricted maximum likelihood estimation with random intercept for subjects was used for analysis of change in Kidscreen-27 scores over the three time points (Tests 1–3). Individual variability was described by presenting mean differences ± SDs and 95 % limits of agreement; Bland Altman plots were used to graphically display the variation (Tests 1–3).

The structure validity of the questionnaire was tested using confirmatory factor analysis (CFA). The overall model fit was assessed using the chi-square test statistic. However as this test is highly sensitive to sample size, we used alternative fit indices having the following cuts-off suggesting acceptable fit; the root mean squared error of approximation (RMSEA), (<0.08); the comparative fit index (CFI) (>90) and the Tucker-Lewis index (TLI) ((>90), while factor loadings should be > 0.40 [7].

For convergent validity, we used the Spearman rank correlation (r_s_) to test whether the Kidscreen-27 scores were positively correlated with the Cantrils Ladder score. Based on previous research, we hypothesized that the Kidscreen-27 psychological well-being domain would be the one most strongly correlated with the Cantrils Ladder score [1, 2]. Correlation coefficients <0.1 were considered trivial, 0.1 – 0.29 as small, 0.30 – 0.49 as moderate, and ≥0.5 as high [4].

The software Prism version 6.05 was used to calculate and display the results of the mean differences with 95 % limits of agreement and the Bland Altman plots. The CFA analyses were conducted with Stata version 14. Other statistical analyses were performed using IBM SPSS version 21. Two-sided *P*-values <0.05 were considered statistically significant.

## Results

**Fig 1:**
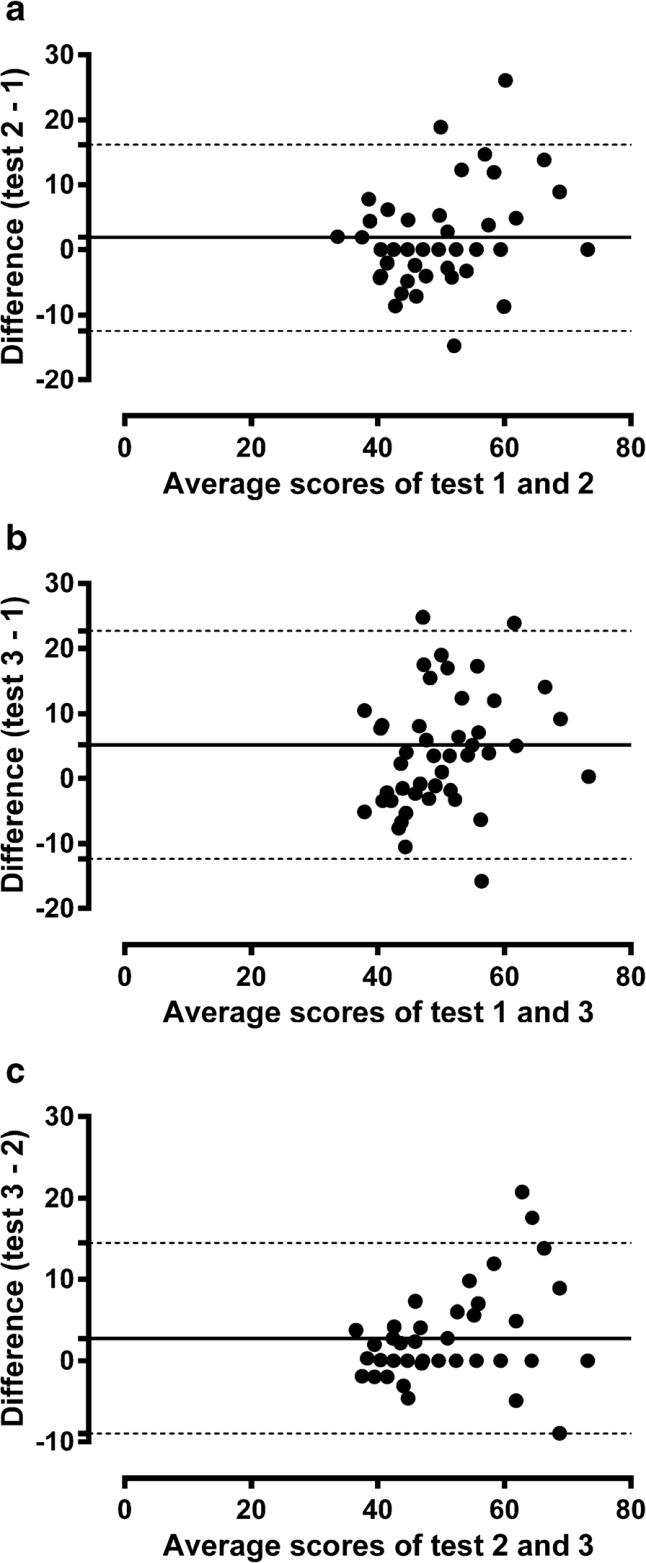
Bland Altman plots showing differences between: Tests 1 and 2 (**a**); 1 and 3 (**b**); and 2 and 3 (**c**), as a function of the mean of the corresponding tests on the Kidscreen-27 physical well-being domain

A total of 1085 children (52.5 % boys) participated in the study (85 % response rate). Table 1 shows the Kidscreen-27 mean *T*-value scores, the percentages of children who had floor and ceiling scores, and Cronbach’s alpha values. ICC calues were as follows; physical well-being (ICC = 0.73), psychological well-being (ICC = 0.72), autonomy & parents (ICC = 0.71), social support & peers (ICC = 0.81) and school environment (ICC =0.79). Floor effects ranged from 0 % to 0.3 % while ceiling effects ranged from 6.3 % to 17.1 %. Trends in the domain scores over time are shown in Table 2. Physical well-being, psychological well-being and autonomy & parents significantly improved over time. The variability over time in the five Kidscreen-27 domain scores is shown in Table 3. By way of an illustrative example, Fig. 1 shows Bland Altman plots for the physical well-being domain.

**Table 1:** Kidscreen-27 *T*-value domain scores, floor and ceiling effects, and internal consistency

Domains	*T*-value	% Floor	% Ceiling	Cronbach’s alpha
Physical well-being (*N* = 1063)	51.1 ± 10.2	0.1	7.3	0.80
Psychological well-being (*N* = 1067)	52.9 ± 9.4	0	8.0	0.82
Autonomy & parents (*N* = 1044)	50.4 ± 9.5	0	6.3	0.80
Social support & peers (*N* = 1081)	51.2 ± 9.6	0.3	17.1	0.79
School environment (*N* = 1085)	53.6 ± 9.7	0.3	12.1	0.77

Note. Variables are means ± SD

**Table 2:** Time trends for changes in the Kidscreen-27 domain scores

Domains	Change (Test 2–1)	Change (Test 3–1)	*P* for trend
Physical well-being (*N* = 55)	2.1 (0.1, 4.1)	5.3 (3.3, 7.3)	<0.001
Psychological well-being (*N* = 56)	2.5 (0.5, 4.5)	2.3 (0.3, 4.4)	0.025
Autonomy & parents (*N* = 55)	1.1 (-0.8, 3.1)	3.8 (1.8, 5.8)	<0.001
Social support & peers (*N* = 56)	-0.9 (-2.3, 0.6)	-1.4 (-2.9, 0.1)	0.188
School environment (*N* = 56)	0.6 (-1.1, 2.4)	1.1 (-0.7, 2.9)	0.480

Note: The results are from Tests 1 - 3. The scores are presented as mean change values and 95 % CIs

**Table 3:** Variation in the Kidscreen-27 scores over time

Domains	Differences ± SD	95 % limits of agreement
Physical well-being		
Test 2 - 1 (*N* = 52)	1.9 ± 7.3	-12.4, 16.2
Test 3 - 1 (*N* = 51)	5.4 ± 8.6	-11.47, 22.3
Test 3 - 2 (*N* = 49)	2.8 ± 6.0	-8.9, 14.5
Psychological well-being		
Test 2 - 1 (*N* = 56)	2.5 ± 7.1	-11.4, 16.3
Test 3 - 1 (*N* = 51)	2.2 ± 8.5	-14.5, 18.7
Test 3 - 2 (*N* = 51)	-0,1 ± 6.9	-13.6, 13.4
Autonomy & parents		
Test 2 - 1 (*N* = 54)	1.2 ± 5.3	-9.1, 11.5
Test 3 - 1 (*N* = 50)	3.8 ± 8.3	-12.4, 19.9
Test 3 - 2 (*N* = 50)	2.5 ± 7.7	-12.7, 17.6
Social support & peers		
Test 2 - 1 ((*N* = 56)	-0.9 ± 4.9	-10.5, 8.7
Test 3 - 1 (*N* = 53)	-1.4 ± 5.9	-13.0, 10.2
Test 3 - 2 (*N* = 53)	-0.6 ± 8.5	-12.1, 10.9
School environment		
Test 2 - 1 (*N* = 55)	0.6 ± 4.4	-8.1, 9.3
Test 3 - 1 (*N* = 50)	1.08.4	-15.4, 17.4
Test 3 - 2 (*N* = 49)	0.4 ± 6.8	-12.9, 13.8

Note: The results are from Test 1 - 3

The CFA (*N* = 938 with complete data on all Kidscreen-27 domains) showed that the chi-square test was statistically significant (*X*^2^ = 974; *df* = 314; *P* <0.001), while RMSEA = 0.046 (90 % CI, 0.044 – 0.051, CFI = 0.93 and TLI = 0.92. The modification indices showed that the model was improved by allowing error covariance between items 6 and 7 on the autonomy & parents scale and between items 4, 5 and 6 on the psychological well-being scale (modified model: *X*^2^ = 707; *df* = 310; *P* <0.001, RMSEA = 0.037 (90 % CI, 0.033 – 0.041, CFI = 0.96 and TLI = 0.95. All factor loading were > 0.40 (Table 4) (see additional file 1 for more CFA details).

**Table 4:** Confirmatory factor analysis of the Kidscreen-27

Measurements	Stand coeff.
Cov. (Physical well-being, Psychological well-being)	0.67
Cov. (Physical well-being, Autonomy & parents)	0.48
Cov. (Physical well-being, Social support & peers)	0.51
Cov. (Physical well-being, School environment)	0.56
Cov. (Psychological well-being, Autonomy & parents)	0.72
Cov. (Psychological well-being, Social support & peers)	0.74
Cov. (Psychological well-being, School environment)	0.80
Cov. (Autonomy & parents, Social support & peers)	0.63
Cov. (Autonomy & parents, School environment)	0.64
Cov. (Social support & peers, School environment)	0.66
Physical well-being	
1. In general, how would you say your health is?	0.55
2. Have you felt fit and well?	0.74
3. Have you been physically active (e. g. running, climbing, biking)?	0.64
4. Have you been able to run well?	0.70
5. Have you felt full of energy?	0.70
Psychological well-being	
1. Has your life been enjoyable?	0.74
2. Have you been in a good mood?	0.71
3. Have you had fun?	0.73
4. Have you felt sad?	0.47
5. Have you felt so bad that you didn’t want to do anything?	0.48
6. Have you felt lonely?	0.53
7. Have you been happy with the way you are?	0.61
Cov. error terms (item 4, 5)	0.27
Cov. error terms (item 4, 6)	0.23
Cov. error terms (item 5, 6)	0.24
Autonomy & parents	
1. Have you had enough time for yourself?	0.48
2. Have you been able to do the things that you want to do in your free time?	0.68
3. Have your parent(s) had enough time for you?	0.71
4. Have your parent(s) treated you fairly?	0.61
5. Have you been able talk to your parent(s) when you wanted to?	0.65
6. Have you had enough money to do the same things as your friends?	0.57
7. Have you had enough money for your expenses?	0.51
Cov. Error term (item 6, 7)	0.39
Social support & peers	
1. Have you spent time with your friends?	0.62
2. Have you had fun with your friends?	0.76
3. Have you and your friends helped each other?	0.73
4. Have you been able to rely on your friends?	0.64
School environment	
1. Have you been happy at school?	0.78
2. Have you got on well at school?	0.75
3. Have you been able to pay attention?	0.62
4. Have you got along well with your teachers?	0.56

Note: 938 children had complete data. Cov: covariance. All standardized coefficient were statistically significant (*P* < 0.001)

The Kidscreen-27 scores were positively and significantly correlated with the Cantrils Ladder score (55 – 56 paired observations). The correlations for the various Kidscreen-27 domains were as follows; physical well-being, r_s_ = 0.29 (*P* = 031); psychological well-being, r_s_ = 0.59 (*P* <0.001); autonomy & parents, r_s_ = 0.31 (‘ 0.025); social support & peers, r_s_ = 0.53 (*P* < 0.001); and school environment, r_s_ = 0.48 (*P* < 0.001).

## Discussion

The findings of this study demonstrate that the reliability and validity of the Norwegian version of the Kidscreen-27 are good. A large body of research findings regarding the Kidscreen-27 enables a direct comparison with our results [1, 2]. Cronbach alpha values in the current study ranged from 0.73 to 0.83. This compares to 0.80 – 0.84 reported in the literature when the instrument was administered to children aged between 8 and 18 years [2]. We found almost no floor effects, but a moderate ceiling effect in the social support & peers domain. These results are similar to previously reported findings [2]. The ICC values in the current study ranged from 0.71 to 0.80, which were slightly higher than the range of 0.61 – 0.66 reported previously [1]. This finding may be a consequence of different test-retest intervals (1 day vs. 2 weeks). Additionally, our study showed variability in Kidscreen-27 scores over time in some individuals. This has been reported with other HRQoL measures and in various populations [8, 9]. Although it could be a consequence of measurement error, natural fluctuations in HRQoL could also be responsible [10]. Previous research has suggested that there is a small retest effect, in order that Kidscreen-27 scores tend to rise if assessed multiple times, even without any interventions [2]. Our study demonstrated this in three of the domains, even though the test-retest intervals were short. Whether this effect is still present if the test-retest interval is longer, is unknown. However, it suggests that studies using Kidscreen-27 to assess the effectiveness of interventions must have a control group.

The CFA showed an acceptable overall model fit, especially when the largest modification indices were taken into account. We think the added covariation terms make sense conceptually. Item 6 and 7 on the autonomy & parents scale are related to perceived family economy while the other items on that scale are not. The questions on item 4, 5 and 6 on the psychological well-being are worded “negatively” such as “Have you felt sad? “(item 4), while item 1, 2, 3 and 7 are worded “positively such as” Has your life been enjoyable?”(item 1). Finally, we found that all the Kidscreen-27 domains were significantly associated with general life satisfaction as measured with the Cantrils Ladder. The sizes of the correlation coefficients are similar to those that have been reported previously when assessing convergent validity using a range of instruments and in various populations [1, 2].

This study was limited as only children aged 10 years were included. It is more usual to assess a wider age group. Our decision to do so was a pragmatic one, as we required a validated instrument to assess HRQoL in 10-year-olds, for the purpose of a randomized controlled trial. However, we propose that if good reliability and validity levels of an assessment instrument are demonstrated in large sample of younger children, it is possible that this will also be the case in older children and adolescents as they typically have better cognitive skills. The variability in Kidscreen-27 scores in children and adolescents are also quite similar [2]. However, because cognitive skills are not the only aspects to consider in the assessment of the psychometric properties of HRQoL instruments, especially in pediatric, pre-adolescent and adolescent individuals, the properties of this instrument should also be investigated in Norwegian children older than 10 years. Our short test–retest intervals might be criticized. However, in addition to the points made above, it should be noted that a previous study found that test-retest effects were similar whether the interval is 2 days or 2 weeks [11].

## Conclusions

Our findings suggest that the Kidscreen-27 works well in Norwegian context, and has good reliability and validity. Further large studies are needed to assess the Kidscreen-27 more fully with regards to its clinical and research utility, and its ability to detect changes in HRQoL following interventions.

## Additional file

Additional file 1: Confirmatory factor analysis conducted with Stata version 14. Page 1–4: results of model 1. Page 4–6: modification indices of model 1. Page 6–9: results of the model 2 (modified model). (PDF 330 kb)

## Abbreviations

CFA: confirmatory factor analysis
CFI: comparative fit index
HRQoL: health-related quality of life
ICC: intraclass correlation
RMSEA: root mean squared error of approximation
TLI: tucker-Lewis index
