# Supplementary material for: Psychometric properties of the Norwegian version of the Kidscreen-27 questionnaire
Source: Health Qual Life Outcomes. 2016 Apr 9;14:58. doi: 10.1186/s12955-016-0460-4 (PMC4826483; doi:10.1186/s12955-016-0460-4)
Supplement: Additional file 1: — Confirmatory factor analysis conducted with Stata version 14. Page 1–4: results of model 1. Page 4–6: modification indices of model 1. Page 6–9: results of the model 2 (modified model). (PDF 330 kb) [file 12955_2016_460_MOESM1_ESM.pdf]

```
                                          ___  ____  ____  ____  ____(R)
                                         /__    /   ____/   /   ____/
                                        ___/   /   /___/   /   /___/
                                          Statistics/Data Analysis
```

```
  ___  ____  ____  ____  ____ (R)
 /__    /   ____/   /   ____/
___/   /   /___/   /   /___/   14.0   Copyright 1985-2015 StataCorp LP
  Statistics/Data Analysis            StataCorp
                                      4905 Lakeway Drive
     Special Edition                  College Station, Texas 77845 USA
                                      800-STATA-PC        http://www.stata.com
                                      979-696-4600        stata@stata.com
                                      979-696-4601 (fax)

40-user Stata network perpetual license:
       Serial number:  401406001559
         Licensed to:  Helse Vest
                       Helse Vest

Notes:
      1.  Unicode is supported; see help unicode advice.
      2.  Maximum number of variables is set to 5000; see help set maxvar.

1 . use "L:\JR.dta", clear

2 . sem (PHYSICAL -> kY27PHY1 kY27PHY2 kY27PHY3 kY27PHY4 kY27PHY5) (PSYCHOLOGICAL -> kY27PWB1 k
> Y27PWB7) (AUTONOMY -> kY27PAR1 kY27PAR2 kY27PAR3 kY27PAR4 kY27PAR5 kY27PAR6 kY27PAR7) (SOCI
> CHOOL -> kY27SCH1 kY27SCH2 kY27SCH3 kY27SCH4),  stand
note: The following observed variable names will be treated as latent variables: KY27PHYc, KC
      PWmiss, KC27pw_T, KC27pa_R, PARmiss, KC27pa_T, KC27pe_R, PERmiss, KC27pe_T, KC27sc_R, S
      intention use the nocapslatent option, or identify the latent variable names in the lat
(306 observations with missing values excluded)

Endogenous variables

Measurement:  kY27PHY1 kY27PHY2 kY27PHY3 kY27PHY4 kY27PHY5 kY27PWB1 kY27PWB2 kY27PWB3 kY27PWE
              kY27PAR2 kY27PAR3 kY27PAR4 kY27PAR5 kY27PAR6 kY27PAR7 kY27SOC1 kY27SOC2 kY27SOC
              kY27SCH4

Exogenous variables

Latent:       PHYSICAL PSYCHOLOGICAL AUTONOMY SOCIAL SCHOOL

Fitting target model:

Iteration 0:   log likelihood = -29053.578
Iteration 1:   log likelihood =   -29019.1
Iteration 2:   log likelihood = -29017.244
Iteration 3:   log likelihood = -29017.224
Iteration 4:   log likelihood = -29017.224

Structural equation model                       Number of obs     =        938
Estimation method  = ml
Log likelihood     = -29017.224

 ( 1)  [kY27PHY1]PHYSICAL = 1
 ( 2)  [kY27PWB1]PSYCHOLOGICAL = 1
 ( 3)  [kY27PAR1]AUTONOMY = 1
 ( 4)  [kY27SOC1]SOCIAL = 1
 ( 5)  [kY27SCH1]SCHOOL = 1
```

Standardized	Coef.	OIM Std. Err.	z	P>|z|	[95% Conf.	Interval]
**Measurement**						
kY27PHY1 <-						
PHYSICAL	.5470555	.0264973	20.65	0.000	.4951218	.5989892
_cons	4.094539	.100014	40.94	0.000	3.898516	4.290563
kY27PHY2 <-						
PHYSICAL	.7458389	.0192404	38.76	0.000	.7081285	.7835494
_cons	3.8748	.0952331	40.69	0.000	3.688147	4.061453
kY27PHY3 <-						
PHYSICAL	.6392676	.0232909	27.45	0.000	.5936184	.6849168

_cons	3.639534	.0901497	40.37	0.000	3.462844	3.816224
kY27PHY4 <-						
PHYSICAL	.7021579	.0208745	33.64	0.000	.6612447	.7430711
_cons	4.043687	.098905	40.88	0.000	3.849837	4.237537
kY27PHY5 <-						
PHYSICAL	.6998409	.0208952	33.49	0.000	.658887	.7407948
_cons	4.289075	.1042696	41.13	0.000	4.08471	4.49344
kY27PWB1 <-						
PSYCHOLOGICAL	.7370935	.017624	41.82	0.000	.702551	.7716359
_cons	4.76785	.1148198	41.52	0.000	4.542807	4.992892
kY27PWB2 <-						
PSYCHOLOGICAL	.7039837	.0191043	36.85	0.000	.66654	.7414275
_cons	5.061932	.1213445	41.72	0.000	4.824101	5.299763
kY27PWB3 <-						
PSYCHOLOGICAL	.7211025	.0183502	39.30	0.000	.6851368	.7570683
_cons	5.538671	.1319787	41.97	0.000	5.279998	5.797345
kY27PWB4 <-						
PSYCHOLOGICAL	.5156469	.0264094	19.53	0.000	.4638853	.5674084
_cons	6.218415	.1472359	42.23	0.000	5.929838	6.506992
kY27PWB5 <-						
PSYCHOLOGICAL	.5227136	.0261519	19.99	0.000	.4714569	.5739703
_cons	6.577886	.1553396	42.35	0.000	6.273426	6.882346
kY27PWB6 <-						
PSYCHOLOGICAL	.5667765	.0246409	23.00	0.000	.5184813	.6150717
_cons	6.301413	.149105	42.26	0.000	6.009172	6.593653
kY27PWB7 <-						
PSYCHOLOGICAL	.6161194	.0226628	27.19	0.000	.5717012	.6605376
_cons	5.059046	.1212804	41.71	0.000	4.821341	5.296752
kY27PAR1 <-						
AUTONOMY	.4752028	.0284256	16.72	0.000	.4194896	.530916
_cons	3.363108	.0842326	39.93	0.000	3.198015	3.528201
kY27PAR2 <-						
AUTONOMY	.6652736	.0220218	30.21	0.000	.6221116	.7084356
_cons	4.109522	.100341	40.96	0.000	3.912858	4.306187
kY27PAR3 <-						
AUTONOMY	.7070188	.0202229	34.96	0.000	.6673827	.7466549
_cons	4.065185	.0993736	40.91	0.000	3.870416	4.259953
kY27PAR4 <-						
AUTONOMY	.6070469	.0240225	25.27	0.000	.5599637	.6541301
_cons	4.648444	.1121795	41.44	0.000	4.428576	4.868311
kY27PAR5 <-						
AUTONOMY	.6441093	.0227089	28.36	0.000	.5996007	.6886178
_cons	4.76785	.1148198	41.52	0.000	4.542808	4.992893
kY27PAR6 <-						
AUTONOMY	.6205332	.0238701	26.00	0.000	.5737486	.6673177
_cons	3.527677	.0877475	40.20	0.000	3.355695	3.699659
kY27PAR7 <-						
AUTONOMY	.5684733	.0258113	22.02	0.000	.517884	.6190625
_cons	4.036609	.0987508	40.88	0.000	3.843061	4.230157
kY27SOC1 <-						
SOCIAL	.6242649	.0239466	26.07	0.000	.5773305	.6711994
_cons	4.515678	.1092506	41.33	0.000	4.301551	4.729805
kY27SOC2 <-						
SOCIAL	.7631622	.0190438	40.07	0.000	.7258371	.8004874

_cons	5.567785	.1326301	41.98	0.000	5.307835	5.827735
kY27SOC3 <-						
SOCIAL	.7286866	.020213	36.05	0.000	.6890699	.7683033
_cons	4.421022	.107167	41.25	0.000	4.210979	4.631066
kY27SOC4 <-						
SOCIAL	.6354352	.0233856	27.17	0.000	.5896003	.6812701
_cons	4.393691	.1065662	41.23	0.000	4.184825	4.602557
kY27SCH1 <-						
SCHOOL	.7796608	.017593	44.32	0.000	.7451791	.8141424
_cons	3.811582	.0938633	40.61	0.000	3.627613	3.995551
kY27SCH2 <-						
SCHOOL	.7488888	.0189001	39.62	0.000	.7118453	.7859324
_cons	4.5688	.1104216	41.38	0.000	4.352377	4.785222
kY27SCH3 <-						
SCHOOL	.6178492	.0240589	25.68	0.000	.5706945	.6650038
_cons	5.21387	.1247267	41.80	0.000	4.96941	5.45833
kY27SCH4 <-						
SCHOOL	.5638017	.025668	21.97	0.000	.5134933	.6141102
_cons	4.582689	.110728	41.39	0.000	4.365667	4.799712
var(e.kY27PHY1)	.7007303	.0289909			.6461518	.7599188
var(e.kY27PHY2)	.4437243	.0287005			.390892	.5036973
var(e.kY27PHY3)	.5913369	.0297782			.5357605	.6526785
var(e.kY27PHY4)	.5069743	.0293144			.4526553	.5678116
var(e.kY27PHY5)	.5102227	.0292467			.456003	.5708892
var(e.kY27PWB1)	.4566932	.0259811			.4085075	.5105627
var(e.kY27PWB2)	.5044069	.0268982			.4543489	.5599801
var(e.kY27PWB3)	.4800112	.0264648			.4308454	.5347874
var(e.kY27PWB4)	.7341083	.0272359			.6826216	.7894784
var(e.kY27PWB5)	.7267705	.0273399			.6751131	.7823805
var(e.kY27PWB6)	.6787644	.0279317			.6261688	.7357779
var(e.kY27PWB7)	.6203969	.0279259			.568008	.6776177
var(e.kY27PAR1)	.7741823	.0270159			.7230024	.8289852
var(e.kY27PAR2)	.557411	.0293011			.5028413	.6179028
var(e.kY27PAR3)	.5001244	.0285959			.4471038	.5594326
var(e.kY27PAR4)	.6314941	.0291655			.5768416	.6913246
var(e.kY27PAR5)	.5851232	.029254			.5305062	.6453633
var(e.kY27PAR6)	.6149386	.0296244			.5595328	.6758308
var(e.kY27PAR7)	.6768382	.0293461			.621697	.73687
var(e.kY27SOC1)	.6102933	.029898			.5544196	.6717978
var(e.kY27SOC2)	.4175834	.029067			.3643285	.4786228
var(e.kY27SOC3)	.4690158	.0294578			.4146917	.5304563
var(e.kY27SOC4)	.5962221	.0297201			.5407269	.6574128
var(e.kY27SCH1)	.3921291	.0274331			.3418846	.4497577
var(e.kY27SCH2)	.4391655	.0283082			.3870443	.4983057
var(e.kY27SCH3)	.6182624	.0297296			.562655	.6793655
var(e.kY27SCH4)	.6821276	.0289434			.6276944	.7412811
var(PHYSICAL)	1	.			.	.
var(PSYCHOLOGICAL)	1	.			.	.
var(AUTONOMY)	1	.			.	.
var(SOCIAL)	1	.			.	.
var(SCHOOL)	1	.			.	.
cov(PHYSICAL,PSYCHOLOGICAL)	.6541078	.026233	24.93	0.000	.6026922	.7055235
cov(PHYSICAL,AUTONOMY)	.4849063	.0328322	14.77	0.000	.4205563	.5492563
cov(PHYSICAL,SOCIAL)	.5066011	.0327333	15.48	0.000	.442445	.5707572
cov(PHYSICAL,SCHOOL)	.5596716	.0308457	18.14	0.000	.4992152	.6201281
cov(PSYCHOLOGICAL,AUTONOMY)	.7031186	.0242277	29.02	0.000	.6556331	.7506041
cov(PSYCHOLOGICAL,SOCIAL)	.7377955	.0232452	31.74	0.000	.6922357	.7833553
cov(PSYCHOLOGICAL,SCHOOL)	.7935976	.0207849	38.18	0.000	.75286	.8343352
cov(AUTONOMY,SOCIAL)	.6208162	.0287906	21.56	0.000	.5643875	.6772448
cov(AUTONOMY,SCHOOL)	.6378435	.0278992	22.86	0.000	.5831621	.6925249
cov(SOCIAL,SCHOOL)	.6618825	.0278245	23.79	0.000	.6073476	.7164175

LR test of model vs. saturated: chi2(314) = 974.95, Prob > chi2 = 0.0000

3 . estat gof, stats(all)

Fit statistic	Value	Description
Likelihood ratio		
chi2_ms(**314**)	**974.948**	model vs. saturated
p > chi2	**0.000**	
chi2_bs(**351**)	**9408.677**	baseline vs. saturated
p > chi2	**0.000**	
Population error		
RMSEA	**0.047**	Root mean squared error of approximation
90% CI, lower bound	**0.044**	
upper bound	**0.051**	
pclose	**0.898**	Probability RMSEA <= 0.05
Information criteria		
AIC	**58216.448**	Akaike's information criterion
BIC	**58657.230**	Bayesian information criterion
Baseline comparison		
CFI	**0.927**	Comparative fit index
TLI	**0.918**	Tucker-Lewis index
Size of residuals		
SRMR	**0.038**	Standardized root mean squared residual
CD	**0.998**	Coefficient of determination

4 . estat mindices

Modification indices

	MI	df	P>MI	EPC	Standard EPC
**Measurement**					
kY27PWB4 <-					
PHYSICAL	**6.389**	**1**	**0.01**	**-.1547022**	**-.1204166**
SOCIAL	**3.934**	**1**	**0.05**	**-.1387123**	**-.1150568**
kY27PWB6 <-					
PHYSICAL	**13.603**	**1**	**0.00**	**-.2308383**	**-.1712669**
AUTONOMY	**5.845**	**1**	**0.02**	**.1697317**	**.123267**
kY27PAR2 <-					
PHYSICAL	**27.785**	**1**	**0.00**	**.3491571**	**.1921771**
PSYCHOLOGICAL	**53.255**	**1**	**0.00**	**.5115018**	**.3568259**
SOCIAL	**16.224**	**1**	**0.00**	**.3008454**	**.1764541**
SCHOOL	**7.355**	**1**	**0.01**	**.145124**	**.1214936**
kY27PAR5 <-					
PHYSICAL	**12.868**	**1**	**0.00**	**-.2274182**	**-.1322572**
PSYCHOLOGICAL	**5.349**	**1**	**0.02**	**-.1546519**	**-.1139929**
SCHOOL	**4.090**	**1**	**0.04**	**-.1033705**	**-.0914374**
kY27PAR6 <-					
SOCIAL	**8.148**	**1**	**0.00**	**-.2515958**	**-.1276479**
kY27PAR7 <-					
PSYCHOLOGICAL	**11.567**	**1**	**0.00**	**-.2680458**	**-.1728016**
SOCIAL	**7.204**	**1**	**0.01**	**-.2267539**	**-.1229059**
kY27SOC1 <-					
SCHOOL	**13.225**	**1**	**0.00**	**-.2030899**	**-.1809601**
kY27SOC2 <-					
AUTONOMY	**9.374**	**1**	**0.00**	**-.2135341**	**-.137716**
kY27SOC3 <-					

AUTONOMY	8.641	1	0.00	.2397283	.1314307
SCHOOL	12.057	1	0.00	.2018487	.1719565
kY27SCH1 <-					
PSYCHOLOGICAL	31.539	1	0.00	.6430974	.4178088
SOCIAL	5.306	1	0.02	.2101002	.1147642
kY27SCH2 <-					
PSYCHOLOGICAL	8.565	1	0.00	-.2829343	-.2113758
AUTONOMY	6.475	1	0.01	-.2017918	-.1164295
SOCIAL	9.943	1	0.00	-.247427	-.1554161
cov(e.kY27PHY1,e.kY27PHY2)	5.407	1	0.02	.0528619	.097559
cov(e.kY27PHY1,e.kY27PHY3)	4.117	1	0.04	.0501008	.0769019
cov(e.kY27PHY1,e.kY27PHY5)	3.943	1	0.05	-.041052	-.0788713
cov(e.kY27PHY1,e.kY27PWB3)	3.972	1	0.05	-.0310219	-.0725229
cov(e.kY27PHY1,e.kY27PWB5)	4.092	1	0.04	-.032899	-.0701947
cov(e.kY27PHY1,e.kY27PAR7)	4.776	1	0.03	.0524035	.0770203
cov(e.kY27PHY1,e.kY27SOC2)	5.895	1	0.02	-.0388898	-.0942629
cov(e.kY27PHY1,e.kY27SCH1)	5.520	1	0.02	-.047188	-.0918263
cov(e.kY27PHY1,e.kY27SCH4)	8.508	1	0.00	.0629826	.1027617
cov(e.kY27PHY2,e.kY27PHY3)	7.539	1	0.01	-.0680541	-.1240904
cov(e.kY27PHY2,e.kY27PWB1)	4.137	1	0.04	.033478	.08136
cov(e.kY27PHY2,e.kY27PWB5)	8.859	1	0.00	.0442771	.1122258
cov(e.kY27PHY2,e.kY27SCH4)	5.908	1	0.02	-.0480163	-.0930659
cov(e.kY27PHY3,e.kY27PHY4)	7.506	1	0.01	.0662914	.116154
cov(e.kY27PHY3,e.kY27PWB4)	4.924	1	0.03	-.036972	-.0788895
cov(e.kY27PHY3,e.kY27PWB6)	7.825	1	0.01	-.0473591	-.100172
cov(e.kY27PHY3,e.kY27PAR2)	7.277	1	0.01	.057892	.1002417
cov(e.kY27PHY3,e.kY27PAR5)	8.804	1	0.00	-.0612724	-.1094138
cov(e.kY27PHY3,e.kY27SOC1)	4.013	1	0.05	.041882	.073766
cov(e.kY27PHY3,e.kY27SCH1)	6.087	1	0.01	-.0515201	-.0990538
cov(e.kY27PHY4,e.kY27PAR6)	7.299	1	0.01	.0617346	.1017133
cov(e.kY27PHY5,e.kY27PAR4)	9.524	1	0.00	-.0549043	-.1155974
cov(e.kY27PHY5,e.kY27SCH3)	5.997	1	0.01	-.0372399	-.0921365
cov(e.kY27PWB1,e.kY27PWB2)	9.350	1	0.00	.042234	.1238124
cov(e.kY27PWB1,e.kY27PWB6)	7.962	1	0.00	-.0377655	-.1064644
cov(e.kY27PWB1,e.kY27PAR4)	4.145	1	0.04	.0336698	.0754844
cov(e.kY27PWB1,e.kY27SOC1)	9.559	1	0.00	-.0494056	-.1159768
cov(e.kY27PWB1,e.kY27SCH1)	10.034	1	0.00	.0510981	.1309381
cov(e.kY27PWB1,e.kY27SCH3)	7.440	1	0.01	-.0386768	-.1018938
cov(e.kY27PWB2,e.kY27PWB3)	14.504	1	0.00	.0453574	.1519485
cov(e.kY27PWB2,e.kY27PWB5)	17.893	1	0.00	-.0505189	-.1544607
cov(e.kY27PWB2,e.kY27PWB6)	11.109	1	0.00	-.0401222	-.1230863
cov(e.kY27PWB2,e.kY27PAR2)	5.548	1	0.02	.03494	.0877475
cov(e.kY27PWB2,e.kY27SOC2)	8.385	1	0.00	-.0334859	-.1163078
cov(e.kY27PWB2,e.kY27SOC4)	6.482	1	0.01	.0389707	.0944366
cov(e.kY27PWB3,e.kY27PWB4)	4.242	1	0.04	-.0233292	-.0758157
cov(e.kY27PWB3,e.kY27PWB5)	7.595	1	0.01	-.0316496	-.1016164
cov(e.kY27PWB3,e.kY27PAR2)	10.769	1	0.00	.0466942	.1231421
cov(e.kY27PWB3,e.kY27PAR6)	13.380	1	0.00	-.062214	-.1351219
cov(e.kY27PWB3,e.kY27PAR7)	7.569	1	0.01	-.0453355	-.1002667
cov(e.kY27PWB3,e.kY27SOC1)	6.955	1	0.01	.0365896	.0981522
cov(e.kY27PWB3,e.kY27SOC2)	5.726	1	0.02	.0265641	.0968886
cov(e.kY27PWB3,e.kY27SOC4)	5.807	1	0.02	-.0353859	-.0900459
cov(e.kY27PWB4,e.kY27PWB5)	55.085	1	0.00	.0863886	.2562314
cov(e.kY27PWB4,e.kY27PWB6)	37.756	1	0.00	.071877	.2139081
cov(e.kY27PWB4,e.kY27PAR4)	6.175	1	0.01	-.0367906	-.0870725
cov(e.kY27PWB4,e.kY27SOC3)	4.288	1	0.04	-.0285137	-.0770638
cov(e.kY27PWB5,e.kY27PWB6)	41.365	1	0.00	.0762452	.2241737
cov(e.kY27PWB5,e.kY27PAR6)	4.313	1	0.04	.0368825	.0731095
cov(e.kY27PWB6,e.kY27PAR5)	4.342	1	0.04	.0298623	.074374
cov(e.kY27PWB7,e.kY27SOC2)	4.274	1	0.04	.0278677	.0806758
cov(e.kY27PWB7,e.kY27SCH3)	4.359	1	0.04	-.0312937	-.0747766
cov(e.kY27PAR1,e.kY27PAR2)	14.849	1	0.00	.0962157	.1423638
cov(e.kY27PAR1,e.kY27SOC4)	5.372	1	0.02	-.0576826	-.0823547
cov(e.kY27PAR1,e.kY27SCH1)	5.397	1	0.02	-.0544573	-.0894695
cov(e.kY27PAR2,e.kY27PAR4)	4.942	1	0.03	-.0448884	-.0862109
cov(e.kY27PAR2,e.kY27PAR6)	4.798	1	0.03	-.0525596	-.0855763
cov(e.kY27PAR2,e.kY27PAR7)	5.595	1	0.02	-.0543315	-.0900812
cov(e.kY27PAR2,e.kY27SOC1)	5.578	1	0.02	.0434687	.0874144
cov(e.kY27PAR2,e.kY27SOC3)	5.027	1	0.02	-.0398217	-.0873374

cov(e.kY27PAR2,e.kY27SCH1)	7.471	1	0.01	-.0502949	-.110407
cov(e.kY27PAR3,e.kY27PAR4)	3.915	1	0.05	.0400188	.0793793
cov(e.kY27PAR3,e.kY27PAR5)	20.784	1	0.00	.0890434	.1875004
cov(e.kY27PAR3,e.kY27PAR7)	5.593	1	0.02	-.054287	-.0929594
cov(e.kY27PAR4,e.kY27PAR5)	7.142	1	0.01	.0516204	.1022414
cov(e.kY27PAR5,e.kY27PAR7)	4.575	1	0.03	-.0470401	-.0804318
cov(e.kY27PAR6,e.kY27PAR7)	134.704	1	0.00	.3158119	.4312256
cov(e.kY27PAR6,e.kY27SOC2)	7.812	1	0.01	-.0489904	-.1103179
cov(e.kY27PAR7,e.kY27SOC2)	6.783	1	0.01	-.0442118	-.1013805
cov(e.kY27PAR7,e.kY27SCH4)	8.412	1	0.00	.0663091	.1023519
cov(e.kY27SOC1,e.kY27SOC2)	29.240	1	0.00	.0901639	.250769
cov(e.kY27SOC1,e.kY27SOC4)	22.129	1	0.00	-.0956369	-.1855762
cov(e.kY27SOC2,e.kY27SOC3)	21.518	1	0.00	-.0848208	-.2572863
cov(e.kY27SOC3,e.kY27SOC4)	6.192	1	0.01	.0518344	.1096955
cov(e.kY27SOC3,e.kY27SCH4)	5.092	1	0.02	.0418202	.0853898
cov(e.kY27SCH1,e.kY27SCH3)	34.236	1	0.00	-.1074412	-.2692431
cov(e.kY27SCH2,e.kY27SCH3)	22.578	1	0.00	.0756603	.2060208
cov(e.kY27SCH3,e.kY27SCH4)	11.390	1	0.00	.0597746	.125593

EPC = expected parameter change

```
5 . sem (PHYSICAL -> kY27PHY1 kY27PHY2 kY27PHY3 kY27PHY4 kY27PHY5) (PSYCHOLOGICAL -> kY27PWB1 k
> Y27PWB7) (AUTONOMY -> kY27PAR1 kY27PAR2 kY27PAR3 kY27PAR4 kY27PAR5 kY27PAR6 kY27PAR7) (SOCI
> CHOOL -> kY27SCH1 kY27SCH2 kY27SCH3 kY27SCH4),  cov (e.kY27PWB4*e.kY27PWB5) cov (e.kY27PWB4
> (e.kY27PAR6*e.kY27PAR7) stand
note: The following observed variable names will be treated as latent variables: KY27PHYc, KC
      PWmiss, KC27pw_T, KC27pa_R, PARmiss, KC27pa_T, KC27pe_R, PERmiss, KC27pe_T, KC27sc_R, S
      intention use the nocapslatent option, or identify the latent variable names in the lat
(306 observations with missing values excluded)

Endogenous variables

Measurement:  kY27PHY1 kY27PHY2 kY27PHY3 kY27PHY4 kY27PHY5 kY27PWB1 kY27PWB2 kY27PWB3 kY27PWE
              kY27PAR2 kY27PAR3 kY27PAR4 kY27PAR5 kY27PAR6 kY27PAR7 kY27SOC1 kY27SOC2 kY27SOC
              kY27SCH4

Exogenous variables

Latent:       PHYSICAL PSYCHOLOGICAL AUTONOMY SOCIAL SCHOOL

Fitting target model:   (modified)

Iteration 0:   log likelihood = -29053.578
Iteration 1:   log likelihood =  -28958.94
Iteration 2:   log likelihood = -28892.299
Iteration 3:   log likelihood = -28883.749
Iteration 4:   log likelihood = -28883.691
Iteration 5:   log likelihood = -28883.691

Structural equation model                       Number of obs     =        938
Estimation method  = ml
Log likelihood     = -28883.691

 ( 1)  [kY27PHY1]PHYSICAL = 1
 ( 2)  [kY27PWB1]PSYCHOLOGICAL = 1
 ( 3)  [kY27PAR1]AUTONOMY = 1
 ( 4)  [kY27SOC1]SOCIAL = 1
 ( 5)  [kY27SCH1]SCHOOL = 1
```

Standardized	Coef.	OIM Std. Err.	z	P>|z|	[95% Conf.	Interval]
**Measurement**						
kY27PHY1 <-						
PHYSICAL	.546518	.0265073	20.62	0.000	.4945647	.5984713
_cons	4.094539	.100014	40.94	0.000	3.898516	4.290563
kY27PHY2 <-						
PHYSICAL	.7449378	.0192559	38.69	0.000	.707197	.7826786
_cons	3.8748	.0952331	40.69	0.000	3.688147	4.061453
kY27PHY3 <-						

PHYSICAL	.6404359	.023237	27.56	0.000	.5948923	.6859795
_cons	3.639534	.0901497	40.37	0.000	3.462844	3.816224
kY27PHY4 <-						
PHYSICAL	.7018307	.0208688	33.63	0.000	.6609287	.7427328
_cons	4.043687	.098905	40.88	0.000	3.849837	4.237537
kY27PHY5 <-						
PHYSICAL	.7005367	.020857	33.59	0.000	.6596576	.7414157
_cons	4.289075	.1042696	41.13	0.000	4.08471	4.49344
kY27PWB1 <-						
PSYCHOLOGICAL	.7425038	.017547	42.32	0.000	.7081124	.7768952
_cons	4.76785	.1148198	41.52	0.000	4.542807	4.992892
kY27PWB2 <-						
PSYCHOLOGICAL	.7143063	.0188139	37.97	0.000	.6774317	.7511808
_cons	5.061932	.1213445	41.72	0.000	4.824101	5.299763
kY27PWB3 <-						
PSYCHOLOGICAL	.7291324	.0181594	40.15	0.000	.6935407	.7647241
_cons	5.538671	.1319787	41.97	0.000	5.279998	5.797345
kY27PWB4 <-						
PSYCHOLOGICAL	.4735832	.0277934	17.04	0.000	.4191092	.5280572
_cons	6.218415	.1472359	42.23	0.000	5.929838	6.506992
kY27PWB5 <-						
PSYCHOLOGICAL	.4807617	.0275735	17.44	0.000	.4267187	.5348047
_cons	6.577887	.1553395	42.35	0.000	6.273427	6.882346
kY27PWB6 <-						
PSYCHOLOGICAL	.5312969	.0259534	20.47	0.000	.4804292	.5821647
_cons	6.301413	.149105	42.26	0.000	6.009172	6.593653
kY27PWB7 <-						
PSYCHOLOGICAL	.6173795	.0227211	27.17	0.000	.5728469	.6619121
_cons	5.059046	.1212804	41.71	0.000	4.821341	5.296752
kY27PAR1 <-						
AUTONOMY	.4807451	.0284588	16.89	0.000	.4249669	.5365233
_cons	3.363107	.0842327	39.93	0.000	3.198014	3.5282
kY27PAR2 <-						
AUTONOMY	.6750835	.0219798	30.71	0.000	.632004	.718163
_cons	4.109522	.100341	40.96	0.000	3.912857	4.306187
kY27PAR3 <-						
AUTONOMY	.7169163	.0201718	35.54	0.000	.6773802	.7564523
_cons	4.065184	.0993736	40.91	0.000	3.870415	4.259953
kY27PAR4 <-						
AUTONOMY	.6107	.0241453	25.29	0.000	.5633761	.6580238
_cons	4.648443	.1121795	41.44	0.000	4.428575	4.868311
kY27PAR5 <-						
AUTONOMY	.651469	.0227181	28.68	0.000	.6069424	.6959956
_cons	4.76785	.1148198	41.52	0.000	4.542807	4.992892
kY27PAR6 <-						
AUTONOMY	.5695153	.0256722	22.18	0.000	.5191988	.6198319
_cons	3.527677	.0877475	40.20	0.000	3.355695	3.699659
kY27PAR7 <-						
AUTONOMY	.5104045	.0277494	18.39	0.000	.4560167	.5647923
_cons	4.036609	.0987508	40.88	0.000	3.843061	4.230157
kY27SOC1 <-						
SOCIAL	.6243019	.0239402	26.08	0.000	.57738	.6712238
_cons	4.515678	.1092506	41.33	0.000	4.301551	4.729805
kY27SOC2 <-						

SOCIAL	.7629376	.0190351	40.08	0.000	.7256295	.8002458
_cons	5.567785	.1326301	41.98	0.000	5.307835	5.827735
kY27SOC3 <-						
SOCIAL	.7291783	.0201766	36.14	0.000	.6896329	.7687236
_cons	4.421022	.107167	41.25	0.000	4.210979	4.631066
kY27SOC4 <-						
SOCIAL	.6350459	.0233929	27.15	0.000	.5891966	.6808952
_cons	4.393691	.1065662	41.23	0.000	4.184825	4.602557
kY27SCH1 <-						
SCHOOL	.7789386	.0176625	44.10	0.000	.7443207	.8135565
_cons	3.811582	.0938633	40.61	0.000	3.627613	3.995551
kY27SCH2 <-						
SCHOOL	.7503243	.0188672	39.77	0.000	.7133453	.7873033
_cons	4.5688	.1104216	41.38	0.000	4.352377	4.785222
kY27SCH3 <-						
SCHOOL	.6181606	.0240719	25.68	0.000	.5709805	.6653406
_cons	5.21387	.1247267	41.80	0.000	4.96941	5.45833
kY27SCH4 <-						
SCHOOL	.562715	.0257234	21.88	0.000	.5122981	.6131319
_cons	4.582689	.110728	41.39	0.000	4.365667	4.799712
var(e.kY27PHY1)	.701318	.0289734			.6467694	.7604673
var(e.kY27PHY2)	.4450676	.0286888			.3922455	.5050031
var(e.kY27PHY3)	.5898419	.0297636			.5342983	.6511596
var(e.kY27PHY4)	.5074336	.0292927			.4531498	.5682202
var(e.kY27PHY5)	.5092484	.0292222			.4550772	.5698679
var(e.kY27PWB1)	.4486881	.0260574			.400416	.5027796
var(e.kY27PWB2)	.4897666	.0268778			.4398213	.5453835
var(e.kY27PWB3)	.4683659	.0264812			.4192362	.523253
var(e.kY27PWB4)	.7757189	.0263249			.7258015	.8290695
var(e.kY27PWB5)	.7688682	.0265125			.7186217	.822628
var(e.kY27PWB6)	.7177236	.0275779			.665657	.7738627
var(e.kY27PWB7)	.6188426	.0280551			.5662277	.6763465
var(e.kY27PAR1)	.7688841	.0273628			.7170816	.824429
var(e.kY27PAR2)	.5442623	.0296764			.4890979	.6056486
var(e.kY27PAR3)	.4860311	.028923			.432524	.5461574
var(e.kY27PAR4)	.6270456	.029491			.5718283	.6875948
var(e.kY27PAR5)	.5755881	.0296002			.5204007	.636628
var(e.kY27PAR6)	.6756523	.0292414			.6207036	.7354653
var(e.kY27PAR7)	.7394872	.0283268			.6860006	.7971442
var(e.kY27SOC1)	.6102471	.0298918			.5543848	.6717385
var(e.kY27SOC2)	.4179261	.0290453			.3647055	.4789132
var(e.kY27SOC3)	.468299	.0294246			.4140376	.5296718
var(e.kY27SOC4)	.5967167	.0297112			.5412351	.6578857
var(e.kY27SCH1)	.3932546	.027516			.3428588	.451058
var(e.kY27SCH2)	.4370135	.028313			.3848998	.4961831
var(e.kY27SCH3)	.6178775	.0297606			.5622165	.6790492
var(e.kY27SCH4)	.6833519	.0289499			.628903	.7425148
var(PHYSICAL)	1	.			.	.
var(PSYCHOLOGICAL)	1	.			.	.
var(AUTONOMY)	1	.			.	.
var(SOCIAL)	1	.			.	.
var(SCHOOL)	1	.			.	.
cov(e.kY27PWB4,e.kY27PWB5)	.2709651	.0313604	8.64	0.000	.2094998	.3324303
cov(e.kY27PWB4,e.kY27PWB6)	.2324874	.0322542	7.21	0.000	.1692703	.2957044
cov(e.kY27PWB5,e.kY27PWB6)	.2409621	.0321368	7.50	0.000	.1779752	.303949
cov(e.kY27PAR6,e.kY27PAR7)	.3917683	.0296076	13.23	0.000	.3337384	.4497981
cov(PHYSICAL,PSYCHOLOGICAL)	.6660461	.0260638	25.55	0.000	.614962	.7171302
cov(PHYSICAL,AUTONOMY)	.481117	.0333603	14.42	0.000	.415732	.5465019
cov(PHYSICAL,SOCIAL)	.5067377	.032729	15.48	0.000	.44259	.5708854
cov(PHYSICAL,SCHOOL)	.5595735	.0308489	18.14	0.000	.4991108	.6200362
cov(PSYCHOLOGICAL,AUTONOMY)	.7197018	.0242999	29.62	0.000	.672075	.7673287
cov(PSYCHOLOGICAL,SOCIAL)	.7405352	.0235106	31.50	0.000	.6944553	.7866152
cov(PSYCHOLOGICAL,SCHOOL)	.7970045	.021023	37.91	0.000	.7558002	.8382088
cov(AUTONOMY,SOCIAL)	.6349417	.0285453	22.24	0.000	.5789939	.6908895

cov(AUTONOMY,SCHOOL)	.6381337	.028287	22.56	0.000	.5826921	.6935753
cov(SOCIAL,SCHOOL)	.6616647	.0278346	23.77	0.000	.60711	.7162194

LR test of model vs. saturated: chi2(310) = 707.88, Prob > chi2 = 0.0000

6 . estat gof, stats(all)

Fit statistic	Value	Description
Likelihood ratio		
chi2_ms(310)	707.881	model vs. saturated
p > chi2	0.000	
chi2_bs(351)	9408.677	baseline vs. saturated
p > chi2	0.000	
Population error		
RMSEA	0.037	Root mean squared error of approximation
90% CI, lower bound	0.033	
upper bound	0.041	
pclose	1.000	Probability RMSEA <= 0.05
Information criteria		
AIC	57957.382	Akaike's information criterion
BIC	58417.538	Bayesian information criterion
Baseline comparison		
CFI	0.956	Comparative fit index
TLI	0.950	Tucker-Lewis index
Size of residuals		
SRMR	0.033	Standardized root mean squared residual
CD	0.998	Coefficient of determination

7 .
